# Preparative Separation of Six Rhynchophylla Alkaloids from *Uncaria macrophylla* Wall by pH-Zone Refining Counter-Current Chromatography

**DOI:** 10.3390/molecules181215490

**Published:** 2013-12-12

**Authors:** Qinghai Zhang, Changhu Lin, Wenjuan Duan, Xiao Wang, Aiqin Luo

**Affiliations:** 1 Department of Biochemical Engineering, School of Life Science, Beijing Institute of Technology, Beijing 100081, China; E-Mail: zhqh8@163.com; 2 Guizhou Academy of Testing and Analysis, Guiyang 550002, China; 3 Guizhou Academy of Sciences, Guiyang 550001, China; E-Mail: linchanghu79@sina.com; 4 TCM Process Control Research Center, Shandong Analysis and Test Center, Shandong Academy of Sciences, 19 Keyuan Street, Jinan 250014, China; E-Mail: wxjn1998@126.com

**Keywords:** pH-zone refining counter-current chromatography, alkaloid, *Uncaria rhynchophylla* Wall

## Abstract

pH-Zone refining counter-current chromatography was successfully applied to the preparative isolation and purification of six alkaloids from the ethanol extracts of *Uncaria macrophylla* Wall. Because of the low content of alkaloids (about 0.2%, w/w) in *U. macrophylla* Wall, the target compounds were enriched by pH-zone refining counter-current chromatography using a two-phase solvent system composed of petroleum ether–ethyl acetate–isopropanol–water (2:6:3:9, v/v), adding 10 mM triethylamine in organic stationary phase and 5 mM hydrochloric acid in aqueous mobile phase. Then pH-zone refining counter-current chromatography using the other two-phase solvent system was used for final purification. Six target compounds were finally isolated and purified by following two-phase solvent system composed of methyl *tert*-butyl ether (MTBE)–acetonitrile–water (4:0.5:5, v/v), adding triethylamine (TEA) (10 mM) to the organic phase and HCl (5 mM) to aqueous mobile phase. The separation of 2.8 g enriched total alkaloids yielded 36 mg hirsutine, 48 mg hirsuteine, 82 mg uncarine C, 73 mg uncarine E, 163 mg rhynchophylline, and 149 mg corynoxeine, all with purities above 96% as verified by HPLC Their structures were identified by electrospray ionization-mass spectrometry (ESI-MS) and ^1^H-NMR spectroscopy.

## 1. Introduction

*Uncaria rhynchophylla* Wall is a traditional Chinese herb, which mostly grows in the Guangxi, Guizhou, Yunnan, Sichuan provinces of China. Its major active components are alkaloids which have the anti-hypertensive, anti-arrhythmic, heart muscle reconstruction reversal, sedative, anti-epilepsy, immunoenhancement activity [1,2,3,4,5], and so on. In recent years, rhynchphylla alkaloids have attracted increasing attention due to their notable effects, low toxicity and few adverse reactions [6]. The preparation and activity effects of rhynchphylla total alkaloids have been intensively studied [7]. However, there are few reports about the preparative isolation of large quantities of pure alkaloids. Because of their low content (about 0.2%, w/w) [8], structural instability, similar structure and basicity, it is difficult to separate and purify pure alkaloids from *U. rhynchophylla* by traditional methods such as column chromatography and prep-HPLC, which are time consuming, and usually required multiple chromatography steps. The overall yields of the above methods were poor, because the basic groups in these compounds make them absorb strongly onto the solid supports during separation. Due to the complexity of this herbal composition, low alkaloid content, low overall yield, and its medicinal importance, methods which could be applied for large scale preparation of pure alkaloids are urgently need.

**Figure 1:**
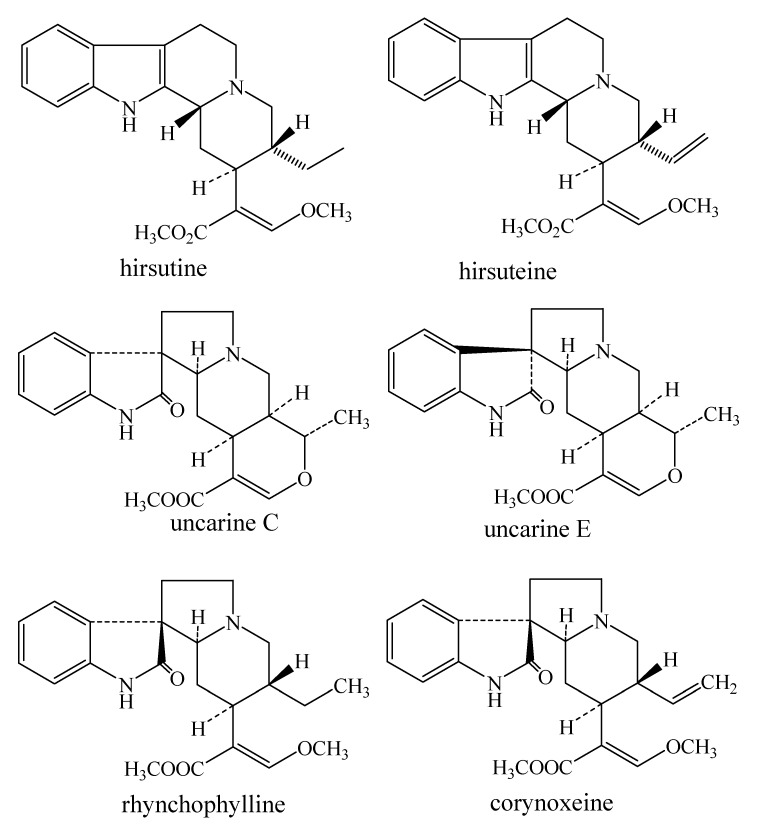
Chemical structures of alkaloids from *U. rhynchophylla* Wall.

pH-Zone-refining counter-current chromatography (CCC) is a modification of HSCCC first introduced by Ito and co-workers [9,10]. It uses a retainer base (or acid) in the stationary phase to retain the analytes in the column and an eluter acid (or base) to elute the analytes according to their pKa values and hydrophobicities, and elutes highly concentrated rectangular peaks fused together with minimum overlapping, while impurities are concentrated and eluted between the outside of the major peaks [11]. It has been identified as a more powerful tool for the separation and purification of alkaloids and organic acids than the conventional CCC method, as if offers an over 10-fold increase in sample loading capacity, high concentration of fractions, concentration of minor impurities, *etc.* This method has been successfully applied to separate and purify a variety of ingredients, including many natural products [12,13,14], peptide derivatives [15,16], synthetic colors [17,18,19] and isomeric ingredients [20,21], but there is no report on the preparative isolation and purification of single alkaloids in *U. rhynchophylla* Wall by pH-zone-refining CCC. In this paper, pH-zone-refining CCC with two different solvent systems was applied to enrich and purify alkaloids from crud extract of *U. rhynchophylla* Wall. Six alkaloids were obtained and their structures were identified by ESI-MS and ^1^H-NMR. The chemical structures of the isolated compounds are shown in Figure 1.

## 2. Results and Discussion

### 2.1. Optimization of HPLC

**Figure 2:**
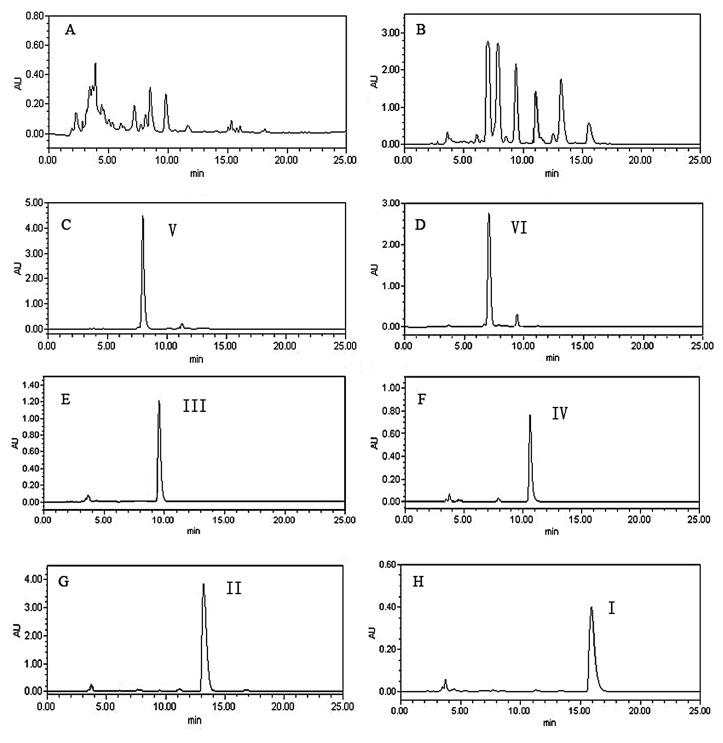
(**A**) HPLC chromatograms of the crude extract; (**B**) HPLC chromatograms of enriched alkaloids; (**C**) HPLC chromatograms of HSCCC peak fraction I; (**D**) HPLC chromatograms of HSCCC peak fraction II; (**E**) HPLC chromatograms of HSCCC peak fraction III; (**F**) HPLC chromatograms of HSCCC peak fraction IV; (**G**) HPLC chromatograms of HSCCC peak fraction V; (**H**) HPLC chromatograms of HSCCC peak fraction VI.

HPLC was used to analyze the crude alkaloids extract from *U. rhynchophylla* Wall and fractions from the pH-zone-refining CCC separation. Several elution systems were tested, such as gradient elution of methanol-water, methanol-TEA solution, methanol-ammonium acetate buffer salt solution. The result showed that suitable separation of the target compounds could be achieved when the mobile phase was composed of A (MeOH) and B (2 mM ammonium acetate solution, adjusted to pH 8.0 with triethylamine) with a gradient elution: 0–30 min, 60%–100% A. The flow rate of the mobile phase was 1.0 mL/min, and the column temperature was maintained at 30 °C. The corresponding HPLC chromatograms are shown in Figure 2.

### 2.2. Selection of Two-Phase Solvent System

Since pH-zone-refining CCC is a liquid-liquid partition separation method, successful separation results are largely dependent upon the selection of a suitable two-phase solvent system, which provides an ideal range of the K values under both acidic (K_acid_ <<1) and basic (K_base_ >> 1) conditions for the target compounds [22,23]. According to the literature, several two-phase solvent systems were tested for the separation of target alkaloids from *Uncaria* rhynchophylla Wall, including ternary system composed of methyl *tert*-butyl ether (MtBE)–acetonitrile–water at different volume ratios and quaternary solvent systems composed of petroleum ether–ethyl acetate–isopropanol–water at different volume ratios.

**Figure 3:**
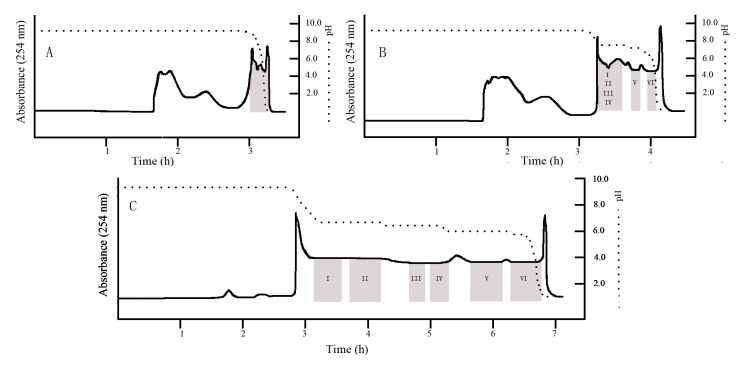
HSCCC chromatogram of crude sample of *U. rhynchophylla* Wall. (**A**) Solvent system: petroleum ether–ethyl acetate–isopropanol–water (2:6:3:9, v/v) with 10 mM TEA in the organic stationary and 5 mM HCl in the aqueous mobile phase; stationary phase: upper phase; mobile phase: lower phase; flow rate: 2.0 mL/min; revolution speed: 800 rpm; retention of stationary phase: 52%; sample size: 2.6 g crude extract; detection at 254 nm; (**B**) Solvent system: MtBE–acetonitrile–water (4:1:5, v/v) with 10 mM TEA in the organic stationary phase and 5 mM HCl in the aqueous mobile phase as two-phase solvent system; stationary phase: upper phase; mobile phase: lower phase; flow rate: 2.0 mL/min; revolution speed: 800 rpm; retention of stationary phase: 48%; sample size: 2.9 g crude extract; detection at 254 nm. (**C**) Solvent system: MtBE–acetonitrile–water (4:0.5:5, v/v) with 10 mM TEA in the organic stationary phase and 5 mM HCl in the aqueous mobile phase; stationary phase: upper phase; mobile phase: lower phase; flow rate: 2.0 mL/min; revolution speed: 800 rpm; retention of stationary phase: 52%; sample size: 2.8 g enriched alkaloids; detection at 254 nm.

After trying the two-phase solvent system composed of petroleum ether–ethyl acetate–isopropanol–water (8:2:1:9, v/v) with 10 mM, TEA in organic stationary phase and 5 mM HCl in the aqueous mobile phase, the results showed that the target alkaloids were eluted with other impurities close to the solvent front. As shown in Figure 3A, when petroleum ether–ethyl acetate–isopropanol–water (2:6:3:9, v/v) with 10 mM TEA in the organic stationary and 5 mM HCl in the aqueous mobile phase were used as two-phase solvent system, the target alkaloids formed a rectangular peak and were separated from other impurities, but the alkaloids were not separated well enough and no pure alkaloid was obtained. Figure 3B shows a pH-zone-refining CCC chromatogram using MtBE–acetonitrile–water (4:1:5, v/v) with 10 mM TEA in the organic stationary phase and 5 mM HCl in the aqueous mobile phase as two-phase solvent system, the separation effect was better than using the two phase solvent system composed of petroleum ether–ethyl acetate–isopropanol–water (2:6:3:9, v/v) with 10 mM TEA in the organic stationary phase and 5 mM HCl in the aqueous mobile phase.

The HPLC analysis results showed that compounds E and F can be isolated from the total alkaloids with high purity, however, comparing with Figure 2A, the separation time is longer (about 4.5 h). Given the observed elution results in Figure 3B, we felt that this solvent system composed of MtBE-acetonitrile-water could be successfully applied to the pH-zone-refining CCC by adjusting the amount of bridge solvent. Therefore, the two phase solvent system composed of MtBE–acetonitrile–water (4:0.5:5, v/v) with 10 mM TEA in the organic stationary phase and 5 mM HCl in the aqueous mobile phase was used to separate alkaloids from *U. rhynchophylla* Wall. The separation result is shown in Figure 3C, and the HPLC analysis results showed that the resolution was improved significantly. Based on the HPLC analysis and the elution curve of the pH-zone-refining CCC chromatogram shown in Figure 3C, all the collected fractions were pooled according to their similar profiles and lyophilized. Retention of the stationary phase was 51.4%, and the total separation time was about 7.5 h. As a result, six alkaloids, including 36 mg of hirsutine, 48 mg of hirsuteine, 82 mg of uncarine C, 73 mg of uncarine E, 163 mg of rhynchophylline, and 149 mg of corynoxeine were obtained from 2.8 g enriched total alkaloids of *Uncaria rhynchophylla* Wall with HPLC purities of 97.8%, 96.1%, 97.5%, 96.9% 97.1% and 96.2%, respectively.

### 2.3. Enrichment Method

The high content of alkaloids in crude extract is essential for the successful separation by pH-zone-refining CCC. Because of the low content of alkaloids in *U. rhynchophylla* Wall, it is difficult to achieve satisfactory content of alkaloids by simple extraction with chloroform. Enrichment by open silica gel column chromatography was tested with chloroform/methanol as eluent, but the enrichment effect was not satisfactory. Because of the adsorption of alkaloids onto the solid support during separation, the selection of silica gel for enrichment is not a good choice. HSCCC is a liquid-liquid partition chromatography without solid support matrix which has many incomparable advantages such as no irreversible adsorption, low risk of sample denaturation, high sample recovery, a large sample loading capa-city, and low cost. Figure 3B showed that after enrichment by pH-zone-refining CCC with the two phase solvent system composed of petroleum ether–ethyl acetate–isopropanol–water (2:6:3:9, v/v) with 10 mM TEA in organic stationary phase and 5 mM HCl in aqueous mobile phase, the content of total alkaloids were improved over 90%.

## 3. Experimental

### 3.1. Apparatus

pH-Zone-refining CCC was carried out using a Model TBE-300A commercial instrument (Shanghai Tauto Biotech Co., Ltd, Shanghai, China), with a multilayer coil of 1.6 mm id and 150 m in length with a total capacity of 300 mL. The *β* values of this preparative column range from 0.5 at internal to 0.8 at the external (*β = r/R*, where r is the rotation radius or the distance from the coil to the holder shaft, and *R* (*R* = 8 cm) is the revolution radius or the distances between the holder axis and central axis of the centrifuge). The solvent was pumped into the column with a Model HX-1050 constant-flow pump (Beijing Bokang Experimental Equipment Co., Ltd, Beijing, China). Continuous monitoring of the effluent was achieved with a Model 8823A-UV Monitor (Beijing Institute of New Technology Application, Beijing, China) at 254nm and a Model 320 pH meter (Mettler Toledo Instruments, Shanghai, China). A manual sample injection valve with 30 mL loop (Shanghai Tauto Biotech Co., Ltd, Shanghai, China) was used to introduce the sample into the column. A portable recorder (Yokogawa Model 3057, Sichuan Instrument Factory, Chongqing, China) was used to draw the chromatogram. The HPLC equipment used was a Shimadzu system including a Shimadzu SPD-20A photodiode array detection (DAD) system, a Shimadzu DGU-20A3 degasser, a Shimadzu CBM-20A communications bus module, a Shimadzu LC-6AD pump, and a Shimadzu workstation (Shimadzu, kyoto, Japan). The identification of pH-zone-refining CCC peak fractions was carried out by ESI-MS on an Agilent 1100/MSD (Agilent Technologies, California, USA,) and by ^1^H-NMR spectra on a Varian-600 NMR spectrometer (Varian, city, state abbrev if USA, country) with CDCl_3_ as solvent and tetramethylsilane (TMS) as internal standard. 

### 3.2. Reagents

Ethanol, methanol, isopropanol, petroleum ether (60–90 °C), ethyl acetate, hydrochloric acid (HCl), and triethylamine (TEA) were all of analytical grade and purchased from Jinan Xinhuicheng Chemical Factory (Jinan, China). Methanol used for HPLC analysis was of chromatographic grade and purchased from Tedia Company, Inc. (Fairfield, OH, USA). Reverse osmosis Milli-Q water (Millipore, Billerica, MA, USA) was used for all solutions and dilutions. The dried twigs of *U. rhynchophylla* Wall were collected from Guizhou Province, China and identified by Jia Li (Shandong University of Traditional Chinese Medicine, Jinan, Shandong, China).

### 3.3. Preparation of Crude Alkaloids

The dried twig of *U. rhynchophylla* Wall (10 kg) was extracted three times with 10 L of 95% ethanol. After filtration, the extracts were combined and evaporated to dryness by rotary evaporation under reduced pressure. Next the residues were dissolved with 1,000 mL water and basified to pH 9.5 with NH_4_OH. After extract with chloroform, 42.6 g crude alkaloids were obtained and used for the subsequent pH-zone-refining CCC experiments.

### 3.4. Preparation of Two-Phase Solvent and Sample Solutions

In this experiment, two different kinds of solvent system were selected to enrich and separate these similar alkaloids. The two-phase solvent system used for enrichment was composed of ether-ethyl acetate–isopropanol–water (2:6:3:9, v/v). The two-phase solvent system used for purification was composed of MtBE–acetonitrile–water (4:0.5:5, v/v). The selected two-phase solvent system was prepared and thoroughly equilibrated by shaking repeatedly. The two phases were separated shortly and degassed by sonication. Then, organic stationary phase was made basic with TEA at the concentration of 10 mM, while the aqueous mobile phase was acidified with HCl at the concentration of 5 mM.

The sample was dissolved in a mixture solution consisting equal volume of organic stationary phase and non-acidified aqueous mobile phase (e.g., 10 mL:10 mL for a 3 g sample portion). Before injecting into column, the solution needed sonicated for several minutes.

### 3.5. Separation Procedure

The separation was initiated by filling the entire column with the organic stationary phase using the pump. Then the sample was injected through the sample injection value. The aqueous mobile was then pumped into the column at 2.0 mL/min while the column was rotated at 850 rpm in the head to tail elution mode. The eluate was continuously monitored the absorbance at 254 nm and collected in test tubes (4 mL/tube). The pH of each eluted fraction was measured with a pH meter. After the separation was completed, retention of the stationary phase was measured by collecting the column contents into a graduated cylinder by forcing them out of the column with pressurized nitrogen gas. The purity of collected fractions was analyzed by HPLC.

### 3.6. HPLC Analyses and Identification of pH-Zone-Refining CCC Peak Fractions

The crude extracts and each purified fraction from the pH-zone-refining CCC separation were analyzed by HPLC with a Shim-pack VP-ODS column (250 × 4.6 mm id) at 241 nm and column temperature of 25 °C. The mobile phase was composed of A (MeOH) and B (2 mM ammonium acetate solution, adjusted to pH8.0 with triethylamine) with a gradient elution: 0–30 min, 60%–100% A. The flow rate of the mobile phase was 1.0 mL/min, and the column temperature was maintained at 30 °C. The effluent was monitored by a diode array detector (DAD).

### 3.7. Identification of Compounds

Identification of alkaloids obtained in the pH-zone-refining CCC was carried out by ESI-MS and ^1^H-NMR as follows:

Compound **1** (peak I in Figure 3C): ESI-MS *m/z*: 369 [M+H]^+^. ^1^H-NMR: 4.45 (1H, d, *J* = 2.4 Hz, H-3), 3.32 (1H, m, H-5), 2.59 (1H, d, *J* = 11.4 Hz, H-6), 3.02 (1H, m, H-6), 7.52 (1H, d, *J* = 7.8 Hz, H-9), 7.13 (1H, t, *J* = 7.8 Hz, H-10), 7.18 (1H, t, *J* = 7.8 Hz, H-11), 7.42 (1H, d, *J* = 7.8 Hz, H-12), 7.33 (1H, s, H-17), 2.04 (1H, m, H-14), 2.47 (1H, m, H-14), 2.22 (1H, m, H-15), 0.77 (3H, t, *J* = 7.2 Hz, H-18), 0.83 (1H, m, H-19), 1.34 (1H, m, H-19), 2.82 (1H, m, H-21), 3.69 (3H, s, -COOCH_3_), 3.78 (3H, s, -OCH_3_). Compared with the data given in reference [24], compound **1** was identified as hirsutine.

Compound **2** (peak II in Figure 3C): ESI-MS *m/z*: 367 [M+H]^+^. ^1^H-NMR: 8.32 (1H,s, NH), 7.52 (1H, d, *J* = 8.4 Hz, H-9), 7.43 (1H, d, *J* = 7.2 Hz, H-12), 7.29 (1H, s, H-17), 7.21 (1H, t, *J* = 7.2 Hz, H-11), 7.14 (1H, t, *J* = 7.2 Hz, H-10), 5.33 (1H, dd, *J* = 10.8, 7.8 Hz, H-19), 5.00 (1H, d, *J* = 16.8 Hz, H-18), 4.95 (1H, d, *J* = 10.2 Hz, H-18), 4.49 (1H, brs, H-3), 3.79 (3H, s, OCH_3_), 3.65 (3H, s, COOCH_3_), 3.32 (2H, m, H-5), 3.04 (1H, dd, *J* = 12.0, 3.6 Hz, H-6), 2.99 (1H, m, H-20), 2.59 (1H, m, H-6), 2.67 (2H, m, H-21), 2.48 (1H, m, H-14), 2.02 (1H, m, H-14). Compared with the data given in reference [24], compound **2** was identified as hirsuteine.

Compound **3** (peak III in Figure 3C): ESI-MS *m/z*: 369 [M+H]^+^. ^1^H-NMR: 2.36 (1H, m, H-3), 2.38 (1H, m, H-5a), 3.30 (1H, d, *J* = 13.2 Hz, H-5b), 1.99 (1H, m, H-6a), 2.40 (1H, m, H-6b), 7.20 (1H, d, *J* = 7.2 Hz, H-9), 7.04 (1H, t, *J* = 7.2 Hz, H-10), 7.18 (1H, t, *J* = 7.8 Hz, H-11), 6.81 (1H, t, *J* = 7.2 Hz, H-12), 1.72 (1H, d, *J* = 13.2 Hz, H-14a), 1.53 (1H, m, H-14b), 2.44 (1H, m, H-15), 7.48 (1H, s, H-17), 1.40 (3H, d, *J* = 6.6 Hz, H-18), 4.55 (1H, q, *J* = 6.6 Hz, H-19), 1.59 (1H, m, H-20), 2.32 (1H, m, H-21a), 3.32 (1H, d, *J* = 13.2Hz, H-21b), 3.60 (3H, s, OCH_3_). Compared with the data given in reference [25], compound **3** was identified as uncarine C.

Compound **4** (peak IV in Figure 3C): ESI-MS *m/z*: 369 [M+H]^+^. ^1^H-NMR: 2.55 (1H, dd, *J* = 12.0, 3.0Hz, H-3), 2.46 (1H, m, H-5a), 3.21 (1H, ddd, *J* = 8.4, 8.4, 2.4 Hz, H-5b), 2.39 (1H, m, H-6b), 1.98 (1H, m, H-6a), 7.25 (1H, d, *J* = 7.2 Hz, H-9), 7.01 (1H, t, *J* = 7.2 Hz, H-10), 7.17 (1H, td, *J* = 7.2, 1.2 Hz, H-11), 6.85 (1H, d, *J* = 7.2 Hz, H-12), 1.61 (1H, m, H-14a), 0.86 (1H, dd, *J* = 12.0, 12.6 Hz, H-14b), 2.50 (1H, m, H-15), 7.40 (1H, s, H-17), 1.40 (3H, d, *J* = 6.6 Hz, H-18), 4.34 (1H, q, *J* = 6.6 Hz, H-19), 1.58 (1H, m, H-20), 2.42 (1H, m, H-21a), 3.27 (1H, dd, *J* = 12.0, 2.4 Hz, H-21b), 3.59 (1H, s, H-23, OCH_3_), 8.40 (1H, brs, NH). Compared with the data given in reference [25], compound **4** was identified as uncarine E.

Compound **5** (peak V in Figure 3C): ESI-MS *m/z*: 385 [M+H]^+^. ^1^H-NMR: 9.10 (1H, s, NH-1), 7.35 (1H, s, H-17), 7.30 (1H, d, *J* = 7.8 Hz, H-9), 7.18 (1H, t, *J* = 7.8 Hz, H-11), 7.05 (1H, t, *J* = 7.8 Hz, H-10), 6.88 (1H, d, *J* = 7.8 Hz, H-12), 3.79 (3H, s, OCH_3_), 3.69 (3H, s, COOCH_3_), 3.24 (1H, m, H-5b), 2.30 (1H, m, H-3), 2.48 (1H, d, *J* = 7.8 Hz, H-5a), 3.30 (1H, dd, *J* = 3.6, 12.0 Hz, H-21b), 2.41 (1H, d, *J* = 7.2 Hz, H-6b), 1.65 (1H, m, H-21a), 1.99 (1H, m, H-14b), 0.97 (1H, m, H-14a), 0.84 (3H, d, *J* = 7.2 Hz, H-18), 2.23 (1H, m, H-15), 1.35 (1H, m, H-19b), 1.06 (1H, m, H-19a), 2.20 (1H, m, H-20). Compared with the data given in reference [26], compound **5** was identified as rhynchophylline.

Compound **6** (peak VI in Figure 3C): ESI-MS *m/z*: 383 [M+H]^+^. ^1^H-NMR: 8.01 (1H, s, 1-NH), 7.30 (1H, s, H-17), 7.28 (1H, d, *J* = 7.8 Hz, H-9), 7.23 (1H, t, *J* = 7.8 Hz, H-11), 7.12 (1H, d, *J* = 7.8 Hz, H-10), 6.92 (1H, d, *J* = 7.8 Hz, H-12), 5.49 (1H, m, H-19), 5.04 (2H, m, H-18), 3.80 (3H, s, OCH_3_), 3.67 (3H, s, COOCH_3_), 3.46 (1H, m, H-3), 3.33 (1H, m, H-15), 3.08 (1H, m, H-20), 2.47–2.61 (3H, m, H-5a, 5b, 21b), 2.37 (1H, d, *J* = 9.6Hz, H-21a), 2.05 (3H, m, H-6a, 6b), 1.92 (1H, m, H-14b), 1.31 (1H, t, *J* = 10.8, H-14a). Compared with the data given in reference [27], compound **6** was identified as corynoxeine.

## 4. Conclusions

In the present study, the pH-zone-refining CCC method was successful used with two different solvent systems to enrich and separate alkaloids from *U. rhynchophylla* Wall. Firstly, by using the pH-zone-refining CCC with biphasic solvent systems composed of petroleum ether-ethyl acetate-isopropanol-water (2:6:3:9, v/v) with 10 mM TEA in the upper phase and 5 mM HCl in the lower phase, the total alkaloids were enriched from 3 g chloroform extract. Then, six pure alkaloids were purified from enriched total alkaloids by pH-zone-refining CCC with two-phase solvent systems composed of MtBE-acetonitrile-water (4:0.5:5, v/v) with 10 mM TEA in the organic stationary phase and 5 mM HCl in the aqueous mobile phase in one step. The results of this research clearly demonstrated that the combined application of the two solvent system of pH-refining CCC can provide a rapid and efficient method for the separation of alkaloids from natural plants.
